# Multi-pathogen infections and Alzheimer’s disease

**DOI:** 10.1186/s12934-021-01520-7

**Published:** 2021-01-28

**Authors:** Dana Vigasova, Michal Nemergut, Barbora Liskova, Jiri Damborsky

**Affiliations:** 1 International Clinical Research Center, St. Anne’s University Hospital Brno, Pekarska 53, 656 91 Brno, Czech Republic; 2 Department of Experimental Biology and RECETOX, Faculty of Science, Loschmidt Laboratories, Masaryk University, Kamenice 5, 625 00 Brno, Czech Republic

**Keywords:** Alzheimer’s disease, Antibacterial, Anti-biofilm, Antifungal, Antiviral, Bacteria, Infectious burden, Parasites, Pathogens, Viruses

## Abstract

Alzheimer’s disease (AD) is a chronic neurodegenerative disease associated with the overproduction and accumulation of amyloid-β peptide and hyperphosphorylation of tau proteins in the brain. Despite extensive research on the amyloid-based mechanism of AD pathogenesis, the underlying cause of AD is not fully understood. No disease-modifying therapies currently exist, and numerous clinical trials have failed to demonstrate any benefits. The recent discovery that the amyloid-β peptide has antimicrobial activities supports the possibility of an infectious aetiology of AD and suggests that amyloid-β plaque formation might be induced by infection. AD patients have a weakened blood–brain barrier and immune system and are thus at elevated risk of microbial infections. Such infections can cause chronic neuroinflammation, production of the antimicrobial amyloid-β peptide, and neurodegeneration. Various pathogens, including viruses, bacteria, fungi, and parasites have been associated with AD. Most research in this area has focused on individual pathogens, with herpesviruses and periodontal bacteria being most frequently implicated. The purpose of this review is to highlight the potential role of multi-pathogen infections in AD. Recognition of the potential coexistence of multiple pathogens and biofilms in AD’s aetiology may stimulate the development of novel approaches to its diagnosis and treatment. Multiple diagnostic tests could be applied simultaneously to detect major pathogens, followed by anti-microbial treatment using antiviral, antibacterial, antifungal, and anti-biofilm agents.
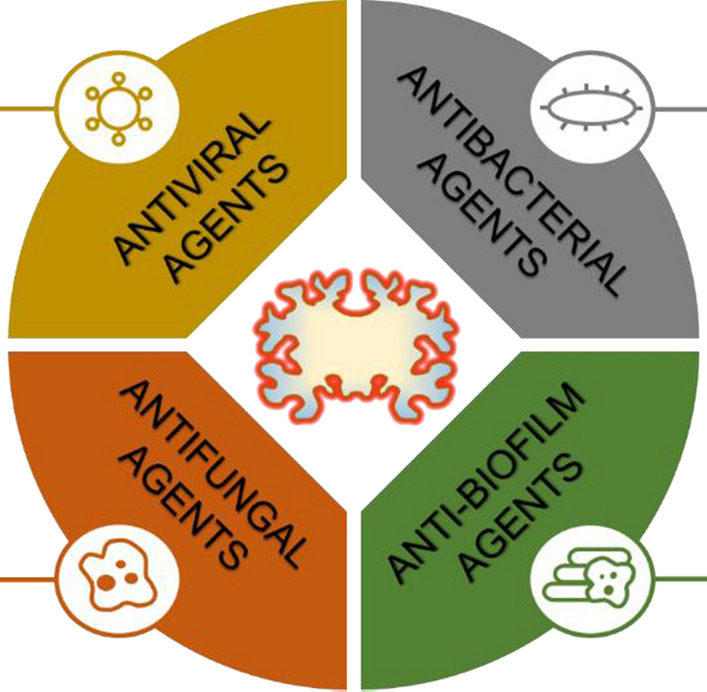

## Introduction

**Fig. 1:**
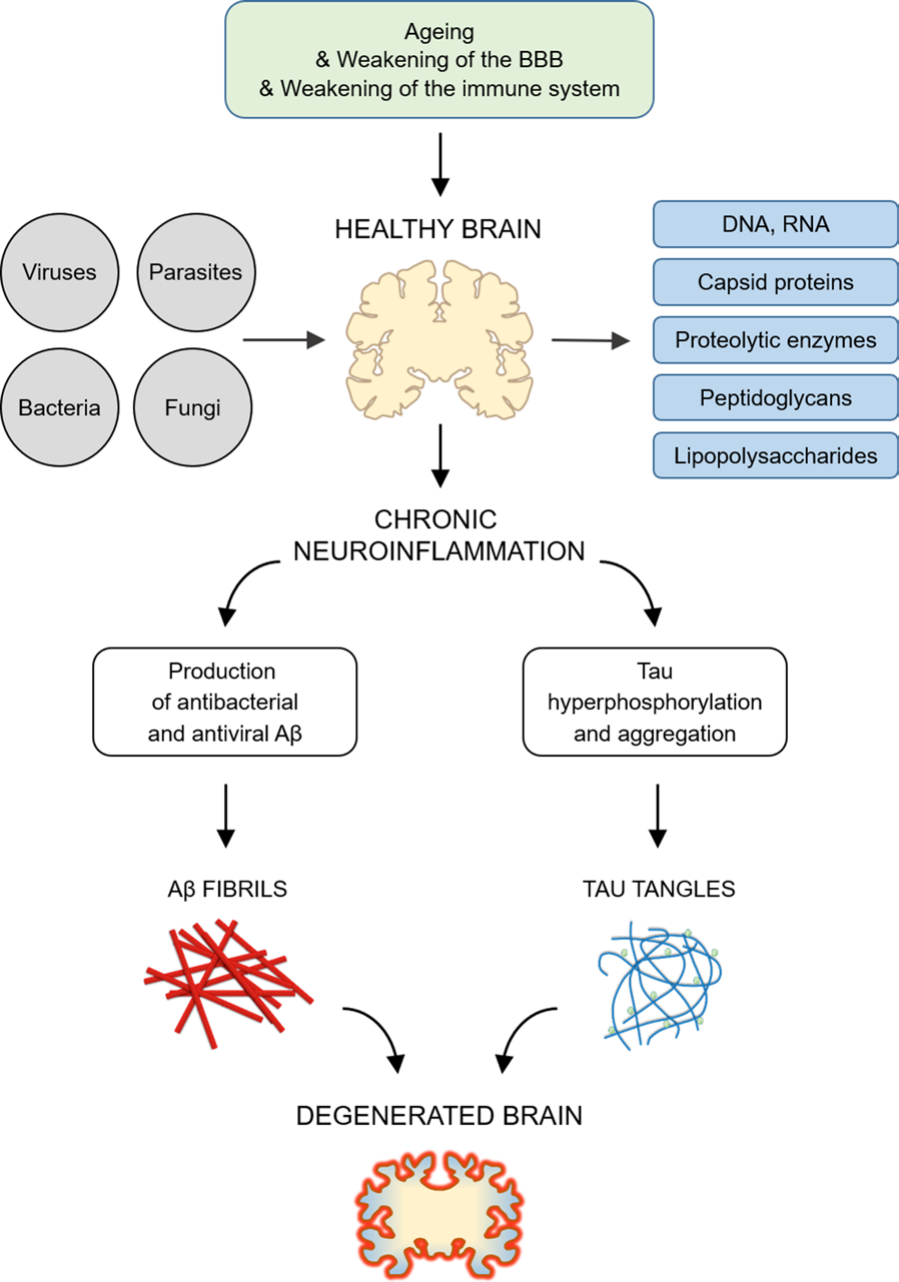
The infection hypothesis of Alzheimer’s disease (AD). Ageing processes leading to increased risks of AD are shown in green. Pathogenic viruses, bacteria, fungi, and parasites potentially associated with AD are shown in grey. Molecular components of pathogenic agents, e.g., DNA, RNA, capsid proteins, proteolytic enzymes, peptidoglycans, and lipopolysaccharides potentially present in biological samples of AD subjects are shown in blue. Two known hallmarks of the disease—Aβ fibrils and Tau tangles—are depicted using red and blue cartoons. Brain tissue, both healthy and degenerated, is represented by yellow cartoons

Alzheimer’s disease (AD) is a progressive brain disorder that destroys memory and thinking skills, ultimately causing an inability to perform even simple tasks. AD causality is multifactorial. The main risk factors include age [1], genetic predisposition [2], cardiovascular disease [3], traumatic brain injury [4], and different environmental factors [5]. The disease is associated with the overproduction and accumulation of amyloid-β peptide and hyperphosphorylation of tau protein in the brain. Although amyloid-β peptide is well known for its neurotoxic potential in AD, there is enough evidence supporting its beneficial roles in protecting the body from infections [6], repairing leaks in the blood–brain barrier [7], promoting recovery from brain injury [8, 9], and regulating synaptic function [10, 11]. In particular, the recent discovery that the amyloid-β peptide has antimicrobial activities strongly supports the possibility of an infectious aetiology of AD and suggests that amyloid-β plaque formation might be induced by infection. The idea that infection may underpin the aetiology of AD was first raised in 1907 [12], and many scientists have since investigated the links between various pathogens and the development of the disease (Fig. 1). Most research in this area has focused on individual pathogens; studies of this type were recently reviewed by Sochocka [13]. However, a growing body of evidence supports the hypothesis of polymicrobial causality [14–20].

**Fig. 2:**
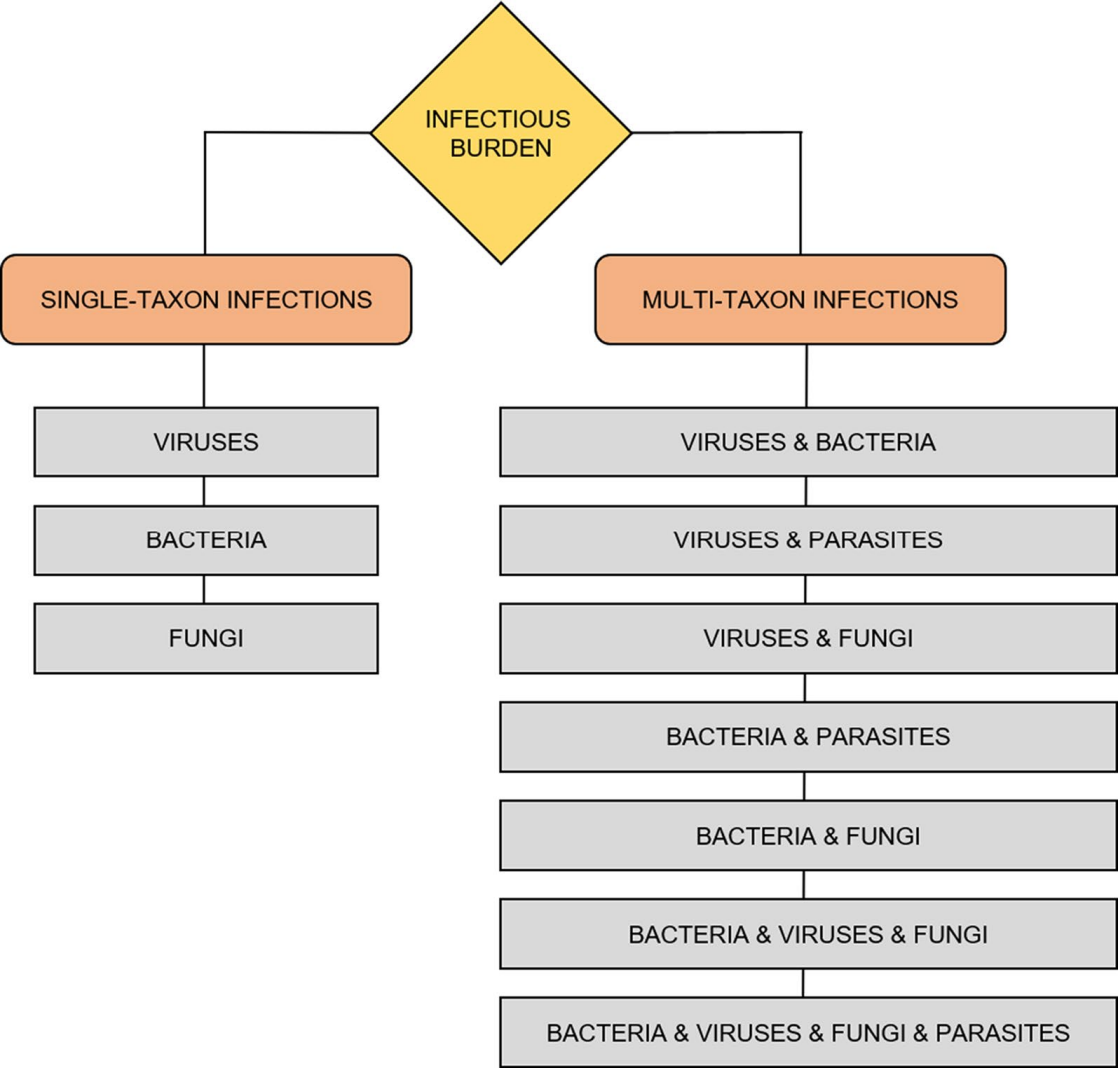
Associations between infectious burden and AD covered by this review. Single-taxon infections are listed in the left column, while combinations of pathogenic taxa that may occur in multi-taxon infections are listed in the right. The infectious burden hypothesis assumes that the combined activity of multiple pathogens contributes to the development of the disease. Most studies on infection and AD have used a limited set of diagnostic tests and could, therefore only examine the contributions of individual taxa. This review highlights the need to use multi-species diagnostic tests in such studies

The multi-microbial or poly-microbial hypothesis has been discussed in terms of the infectious burden and assumes that the collective, cumulative activity of multiple pathogens contributes to the development of disease [21]. Here we summarize this interesting topic, with a particular focus on the roles of herpetic viruses, bacterial and fungal pathogens, and representative parasites (Fig. 2). Other pathogens are also mentioned when appropriate. Articles on cognitive decline and impairment in the context of infectious burden are discussed. The articles for this review were obtained by PubMed searches using the search strategy explicitly described in the Additional file 1. Selected studies identifying single-taxon (Table 1) and multi-taxon (Table 2) pathogens in samples from subjects with AD are systematized. Additionally, potential antimicrobial therapeutic strategies such as treatment with antiviral, antibacterial, antifungal, antiparasitic and anti-biofilm agents are suggested.

**Table 1:** Studies identifying single-taxon pathogens in samples from subjects with AD

Authors, Years	Pathogens	Matrix	Sample size	Methodology	References
**Herpetic viruses**					
Lövheim et al., 2018	HSV-1, CMV	Plasma	360	ELISA	[23]
Lin et al., 2002	HSV-2, CMV, HHV-6	Brain	148	PCR-based analysis	[28]
Carbone et al., 2014	CMV, EBV, HHV-6	Brain Plasma PBL	93	PCR-based analysis, ELISA	[29]
Redhead et al., 2018	HHV-6, HHV-7	Brain	Cohort study	RNA sequencing, statistical analysis	[30]
Hemling et al., 2003	HSV-1, HHV-6, VZV	Brain	34	PCR-based analysis	[31]
**Bacteria**					
Riviere et al., 2002	*Treponema* species	Brain	19	PCR-based analysis, immunochemical analysis	[41]
Kamer et al., 2009	Periodontal bacteria	Plasma	18	ELISA	[42]
Beydoun et al., 2020	*Helicobacter pylori*, periodontal bacteria	Serum	1431	Immunochemical analysis, statistical analysis	[43]
Sparks Stein et al., 2012	Periodontal bacteria	Serum	81	ELISA	[44]
Emery et al., 2017	Actinobacteria, Bacteroidetes, Firmicutes, Proteobacteria	Brain	14	16S rRNA sequencing	[45]
Siddiqui et al., 2019	Gingivitis bacteria Periodontal bacteria	Brain	10	16S rDNA sequencing	[46]
**Fungi**					
Alonso et al., 2014	*Saccharomyces cerevisiae, Malassezia globosa*, *Malassezia restricta, Penicillinum, Phoma*	Serum	29	Immunofluorescence analysis, slot-blot analysis	[47]
Alonso et al., 2014	*Candida* sp., *Saccharomyces cerevisae*, *Rhodotorula mucilaginosa*	Brain	11	Proteomic analysis PCR-based analysis	[48]
Alonso et al., 2015	*Candida albicans*, *Cladosporium cryptococcus, Malasezzia globosa* *Malasezzia restricta, Saccharomyces cerevisiae*	CSF	10	PCR-based analysis, slot-blot analysis	[49]
Pisa et al., 2015	*Candida* sp., *Cladosporium*, *Phoma*, *Malassezia globosa*, *Malassezia restricta*, *Neosartorya hiratsukae*, *Saccharomyces cerevisiae*, *Sclerotina borealis*	Brain	11	PCR-based analysis, immunochemical analysis	[50]
Alonso et al., 2017	*Candida albicans*, *Cladosporium cryptococcus, Malasezzia globosa* *Saccharomyces cerevisiae*	Brain	9	PCR-based analysis, NGS	[51]

*PBL* peripheral blood leukocytes, *CSF* cerebrospinal fluid, *NGS* next-generation sequencing

**Table 2:** Studies describing multi-taxon pathogens and their effect on cognitive impairment

Authors, Years	Pathogens	Matrix	Sample size	Methodology	References
**Viruses and bacteria**					
Bu et al., 2014	HSV-1, CMV, *Borrelia burgdorferi*, *Chlamydia pneumoniae*, *Helicobacter pylori*	Serum	128 AD	ELISA	[15]
Strandberg et al., 2003	HSV-1, HSV-2, CMV, *Chlamydia pneumoniae, Mycoplasma pneumoniae*	Serum	383	ELISA	[52]
Strandberg et al., 2005	HSV-1, HSV-2, CMV, *Chlamydia pneumoniae, Mycoplasma pneumoniae*, *Helicobacter pylori*	Serum	58	ELISA, PCR-based analysis	[53]
Katan et al., 2013	HSV-1, HSV-2, CMV, *Chlamydia pneumoniae*, *Helicobacter pylori*	Serum	1625	ELISA	[54]
Wright et al., 2015	HSV-1, HSV-2, CMV, *Chlamydia pneumoniae, Helicobacter pylori*	Serum	588	ELISA	[55]
Renvoize et al., 1987	HSV-1, CMV, Adenovirus, Influenza A and B, Measles *Chlamydia Group B*, *Coxiella burnettii, Mycoplasma pneumoniae*	Serum	33 AD	Immunochemical analysis	[56]
**Viruses and parasites**					
Gale et al., 2016	CMV, HSV-1, HSV-2 Toxocaris, Toxoplasmosis, Hepatitis A, B, C	Serum	5662	Immunochemical analysis	[66]
Nimgaonkar et al., 2016	HSV-1, HSV-2, CMV, *Toxoplasma gondii*	Serum	1022	Immunochemical analysis	[67]
**Viruses and fungi**					
Kuboshima et al., 2007	CMV, Aspergilloma	Lungs	1 AD	Autopsy	[68]
**Bacteria and parasites**					
Gale et al., 2015	*Helicobacter pylori*, *Toxoplasma gondii*	Serum	1785	Immunochemical analysis	[69]
**Bacteria and fungi**					
Alonso et al., 2018	Several bacterial and fungal species	Brain	10 AD	Immunochemical analysis, PCR-based analysis, NGS	[14]
**Bacteria, viruses, and fungi**					
Pisa et al., 2018	NC	Brain	2 AD	Immunochemical analysis, PCR-based analysis, Proteomic analysis	[19]
**Bacteria, viruses, fungi, and parasites**					
Pisa et al., 2017	NC	Brain	10 AD	Immunochemical analysis, PCR-based analysis	[20]

*NC* not confirmed, *AD* samples from patients with confirmed AD

## Single-taxon infections

### Herpetic viruses

*Herpesviridae* is a family of double-stranded DNA viruses, eight of which are known to infect humans and cause neurological disease: (i) herpes simplex virus 1 (HSV-1), (ii) herpes simplex virus 2 (HSV-2), (iii) varicella zoster virus (VZV), (iv) human cytomegalovirus (CMV), (v) Epstein-Barr virus (EBV), (vi) human herpesvirus 6 (HHV-6), (vii) human herpesvirus 7 (HHV-7), and (viii) human herpesvirus 8 (HHV-8). A notable aspect of their behaviour is that following infection, they can enter a latent phase and potentially become reactivated in the event of immunity impairment [22]. Several studies have provided evidence of associations between various herpetic viruses, a decline in cognitive abilities, and AD. The most studied viruses in this context are HSV-1 and CMV (Table 1). Positive associations between these two viruses were observed in several serological studies [23–25]. Additionally, Watson et al. described an association between cognitive decline and cumulative exposure to CMV, HSV-1, and HSV-2 [26]. Similarly, using a new method for viral DNA amplification from formalin-fixed AD brain tissue, Rodriguez et al. demonstrated the presence of HSV-1 and CMV but not HSV-2 in a limited set of samples [27].

Significant associations have also been observed between HHV-6 and HSV-1 [28], HHV-6 and EBV [29], and HHV-6 and HHV-7 [30]. A PCR-based analysis of AD-infected and control brain samples performed by Lin et al. showed that the proportion of AD samples containing HHV-6 DNA sequences was higher than in controls (70% versus 40%, *p* = 0.003) and that the presence of HHV-6 overlapped strongly with that of HSV-1 in AD samples [28]. In another multiscale statistical analysis of three independent AD cohorts, Readhead et al. demonstrated increased levels of HHV-6A and HHV-7 transcripts in brains of AD patients, as well as increased levels of HSV-1, encoded latency-associated transcripts [30]. Carbone et al. analyzed HHV-6, CMV, and EBV DNA in peripheral blood leukocytes and brain samples together with IgG levels in plasma samples from AD patients and healthy controls [29], revealing increased levels of EBV and HHV-6 DNA in peripheral blood leukocytes as well as increased CMV and EBV IgG levels in patients who developed AD in the following 5 years. However, several other studies found either no evidence of herpetic infection in AD samples or no significant association between more than one of these viruses and AD [31–36].

## Bacteria

One of the first pieces of evidence suggesting the involvement of bacteria in the development of neurological disorders was the discovery of *Treponema pallidum* in the paretic brains of syphilitic patients [37]. Over 70 years later, MacDonald and Miranda reported the presence of another bacterium, *Borrelia burgdorferi*, in the brains of AD patients [38], and Miklossy et al. noted obvious similarities between the clinical and pathological signs of AD and syphilis [39]. Both *T. pallidium* and *B. burgdorferi* bacteria belong to the phylum *Spirochaetes*, which, like the herpetic viruses, has neurotrophic effects and can enter a latent state after initial infection [40].

In addition to spirochetes, the roles of various oral *Treponema* species and other periodontal bacteria in the aetiology of AD have been investigated (Table 1). The presence of several *Treponema* species was detected in different brain regions of AD patients, and multiple species were identified in several cases [41]. The possibility that co-infection by multiple spirochetes might contribute to the development of AD was subsequently raised by Miklossy [39]. Serological studies performed by Kamer et al. further supported the hypothesis that periodontal bacteria might contribute to AD because an AD group exhibited elevated levels of antibodies against *Aggregatibacter actinomycetemcomitans*, *Porphyromonas gingivalis*, and *Tannerella forsythia* [42]. Additionally, Beydoun et al. showed that co-infection with *Helicobacter pylori* and periodontal pathogens may alter the onset of AD [43]*.* Another well-designed serological study monitored levels of antibodies against 7 periodontal bacteria and reported significantly increased antibody levels (α = 0.05) against *Fusobacterium nucleatum* and *Prevotella intermedia* in AD patients [44]. 16S rRNA sequencing analysis of a limited number of AD and control brain samples (frozen and fixed in formaldehyde) revealed a 5–10-fold increase in bacterial reads in AD samples compared to healthy controls [45]. A more recent study also confirmed the presence of bacterial species associated with gingivitis and periodontal disease in AD brain samples [46].

### Fungi

Early studies identified antibodies against various yeast cells, fungal proteins, and (1,3)-β-glucans in AD patients' blood serum [47]. Eleven AD patients from a group of 29 exhibited high immunoreactivity against a majority of tested *Candida* species, and a further two patients exhibited high reactivity towards a single *Candida* species (Table 1). Moreover, very high levels of fungal antigens were detected in 6 of 29 patients with AD, 8 patients exhibited high levels, and 8 patients exhibited high levels of antigens originating from at least one *Candida* spp. and moderate levels of antigens from at least one other species. Strikingly, Fungitell tests indicated that fungal polysaccharides were present in the blood serum of 28 of the 29 AD patients, suggesting that almost all of the patients had a disseminated fungal infection [47]. Follow-up proteomic analyses showed that 4 fungal peptides were present in 3 AD brain samples but not in a control sample [48]. Extraction and sequencing of DNA from 8 AD patients revealed 5 fungal species: *Saccharomyces cerevisiae*, *Malassezia globosa*, *Malassezia restricta*, *Penicillium* and *Phoma*. Multiple species were detected in several individual patients [48]. Slot-blot analysis of cerebrospinal fluid was used to detect antigens in 10 AD samples and 3 controls; fungal antigens were detected in the AD cerebrospinal fluid with high statistical confidence (*p* = 0.0016, odds ratio = 8) [49]. Moreover, DNA analysis and sequencing of 6 AD samples revealed the presence of 6 fungal species: *Candida albicans*, *Cladosporium*, *Cryptococcus*, *Malasezzia globosa*, *Malasezzia restricta* and *Saccharomyces cerevisiae*. Interestingly, 4 of the 6 samples contained multiple fungal species, indicating multi-fungal infection [49].

Pisa et al. investigated the presence of various yeast species in four different brain regions [50]. Immunohistochemical analysis confirmed fungal infection in different brain sections. DNA amplification and sequencing of one AD and one control sample revealed the following species: *Candida albicans*, *Candida ortholopsis*, *Candida tropicalis, Cladosporium*, *Malassezia globosa*, *Malassezia restricta*, *Neosartorya hiratsukae*, *Phoma*, *Saccharomyces cerevisiae* and *Sclerotinia borealis* (Table 1). Some of these species were detected in the same brain region repeatedly, in keeping with previous reports of multi-fungal infections in AD patients [50]. Next-generation sequencing was subsequently used to analyze fungal DNA in samples representing four brain regions from a single AD patient [51], revealing the presence of an impressive array of yeast species. Notably, *Cryptococcus curvatus* and *Botrytis cinerea* were detected in every studied region. Analysis of two brain regions from a healthy control sample also revealed the presence of diverse fungal species. However, the species identified in control differed from those in the AD samples [51].

## Multi-taxon infections

### Viruses and bacteria

The effect of the cumulative viral and bacterial burden on cognition was systematically investigated by Strandberg et al., who tested seropositivity towards HSV-1, HSV-2, CMV, *Chlamydia pneumoniae*, and *Mycoplasma pneumoniae* in an elderly Finnish population (Table 2). The results of this comprehensive study indicated that viral burden was associated with cognitive impairment, but no association with bacterial burden was observed [52]. A follow-up study investigated the presence of *Helicobacter pylori* in addition to the pathogens listed above: seropositivity towards 3 herpetic viruses and 3 bacteria along with APOE ε4 and several other factors was tested in a cohort of 357 elderly Finnish residents. An association between herpetic viruses and cognitive impairment was again observed. Besides, the presence of APOE ε4 and low education were shown to significantly affect cognitive impairment [53].

Katan and colleagues measured levels of antibodies against HSV-1, HSV-2, CMV, *Chlamydia pneumoniae*, and *Helicobacter pylori* in a population of 1625 elderly participants, and observed a positive correlation between infectious burden and cognitive impairment [54]. A similar association was observed even when only the viral infectious burden was considered. Another systematic and well-executed study supporting an association between infectious burden and cognitive functions was published by Wright et al., who demonstrated a strong association between infection with five pathogens (HSV-1, HSV-2, CMV, *Chlamydia pneumoniae*, and *Helicobacter pylori*) and cognitive decline in the memory domain by testing samples from 588 stroke-free participants [55]. Bu et al. tested titers of antibodies against HSV-1, CMV, *Chlamydia pneumoniae*, *Helicobacter pylori*, and *Borrelia burgdorferi* in a cohort of 128 AD patients and 135 controls and showed that the total burden of infection with these species was associated with AD [15]. However, Renvoize et al. investigated serum antibody titers against 9 pathogens and found no significant differences between 33 AD patients and 28 healthy controls [56].

### Viruses and parasites

Infection with the parasitic intracellular protozoan *Toxoplasma gondii* and parasitic *Toxocara* spp. have been reported to be accompanied by viral hepatitis infections (Table 2). *T. gondii* is the main cause of toxoplasmosis and is highly prevalent worldwide [57]. Like herpetic viruses and spirochetes, *Toxoplasma* exhibits strong CNS tropism, is preferentially localized within specific brain regions, and has been linked to various neuropsychiatric disorders [58, 59]. Several murine studies have revealed associations between *Toxoplasma* infection and AD [60, 61], but the results of human studies have been rather inconsistent. One study showed that AD patients exhibited higher levels of antibodies against *T. gondii* than healthy controls [62]. The helminths *Toxocara canis*, *Toxocara cati* and *Taenia solium* are another group of parasites whose role in dementia has been investigated. Infection with *Toxocara* species is a common zoonosis, and is reported to have diverse neurological consequences, including dementia [63, 64]. Infection with *Taenia solium* is also known as cysticercosis; the form affecting the human nervous system is called neurocysticercosis, which causes a range of neuropsychiatric symptoms linked to dementia [65].

Two excellent serological studies have investigated the relationship between viruses, parasites, and cognitive function. The first examined the association between eight pathogens (HSV-1, HSV-2, CMV, HAV, HBV, HCV, Toxocariasis, and Toxoplasmosis) and cognitive decline in a cohort of 5662 young to middle-aged participants. HSV-1, CMV, and HAV were found to be strongly associated with cognitive decline. HSV-2, Toxoplasmosis, Toxocariasis and HBV were also associated with decline, albeit less strongly than the first group. Surprisingly, HCV appeared to be the pathogen with the weakest association [66]. The second study measured levels of antibodies against HSV-1, HSV-2, CMV, and *Toxoplasma gondii* in a cohort of 1022 participants whose cognitive status was monitored over a 5 year follow up period. Interestingly, HSV-2, CMV, and *Toxoplasma gondii* were associated with accelerated cognitive decline, but HSV-1 was not [67].

### Viruses and fungi

Kuboshima et al. reported the admission of a patient with health complications, including AD and depression (Table 2). Despite care and treatment, the patient died on the 26th day of hospitalization. An autopsy revealed a pulmonary aspergilloma infection together with CMV infection throughout the lungs [68].

### Bacteria and parasites

An extensive study using NHANES III data examined interactions between bacteria and parasites and their mutual association with cognitive function by looking at *Helicobacter pylori* and *Toxoplasma gondii* seropositivity among 1785 young and middle-aged adults with ages between 20 and 59 years [69]. The study showed that joint infection by these two pathogens increased susceptibility to cognitive deficits compared to the effect of a single infection. Interestingly, the study also revealed an association between *Helicobacter pylori* seropositivity and some of the other tested factors. In particular, participants with lower levels of education were at greater risk of cognitive deficits than more highly educated seropositive participants. Race-ethnicity also appeared to be an important factor relating to *Helicobacter pylori* seropositivity and cognitive functions [69].

### Bacteria and fungi

The coincidence of bacterial and fungal infections was studied by Alonso et al., who combined immunohistochemical analysis with PCR experiments and next-generation sequencing of different CNS tissues obtained from AD patients, elderly people, and healthy controls [14]. Immunohistochemical analyses showed that numbers of fungal structures were highest in tissues positive for AD, while next-generation sequencing revealed that *Alternaria*, *Botrytis*, *Candida*, and *Malassezia* were the most strongly represented fungal genera (Table 2). Detailed assessments showed *Alternaria* and *Malassezia* to be more prominent in AD samples, while *Aspergillus*, *Candida*, and *Davidiella* dominated in the elderly group and samples from young subjects had the highest levels of *Phoma* and *Botrytis*. Next-generation sequencing of bacterial DNA in CNS tissues revealed that AD patients had higher levels of *Burkholderiaceae* and *Staphylococcaceae* transcripts, whereas *Micrococcaceae*, *Pseudomonadaceae*, *Sphingomonadaceae*, and *Xanthomonadaceae* were more abundant in controls [14].

### Bacteria, viruses and fungi

The multi-pathogen infectious burden due to bacteria, viruses, and fungi was examined by Pisa et al., who searched for fungal, bacterial, and viral proteins in small bodies known as *corpora amylacea* that are commonly observed in the brains of patients with neurological disease [19]. Mass spectrometry analysis was used to identify fungal, bacterial, and viral peptides in *corpora amylacea* fractions from the brains of two AD patients. Additionally, fungal genera were identified by nested PCR. This battery of methods revealed the presence of fungal and bacterial peptides and sequences, but no peptides corresponding to viruses were found in the studied samples [19].

### Bacteria, viruses, fungi and parasites

Pisa et al. tested for the presence of early and latent forms of HSV-1, *Borrelia burgdorferi*, *Chlamydia pneumoniae*, *Candida* species and *Toxoplasma gondii* in 10 brain samples from AD patients using immunohistochemistry and nested PCR [20]. Immunohistochemical analyses revealed the presence of several fungal structures, while PCR analysis followed by sequencing confirmed the presence of several bacterial species (Table 2). However, the simultaneous presence of HSV-1, *Chlamydia pneumoniae*, *Borrelia burgdorferi*, and *Toxoplasma gondii* was not confirmed [20].

## Antimicrobial therapeutic strategies

### General considerations

Several working hypotheses that were proposed to explain the complex origins of AD have served as starting points for drug development. However, none of these efforts has yielded effective treatments, suggesting that the underlying hypotheses may be invalid [70]. The multi-microbial infectious hypothesis merges two previously established AD hypotheses: (i) production of the antimicrobial Aβ peptide as part of an innate immune response [71–74] and (ii) stimulation of neuroinflammation [75, 76]. New therapeutic strategies for AD can be envisioned based on systematic diagnostic testing for multiple pathogens followed by therapy using antiviral, antibacterial, anti-inflammatory, anti-fungal, and anti-biofilm agents (Table 3).

**Table 3:** Antimicrobial agents that have been used to treat patients with AD

Authors, Years	Agents	Participants	Study design	Outcome	References
Tzeng et al., 2018	Acyclovir, Famciclovir, Gangciclovir, Valacyclovir, Valganciclovir	8362	Cohort study	Decreased risk of dementia	[77]
Devanand et al., 2020	Valacyclovir	130	Randomized, double-blind, controlled trial	NA	[81]
Loeb et al., 2004	Rimfapicin, Doxycycline	101	Randomized, triple-blind, controlled trial	Lower cognitive decline	[83]
Kountouras et al., 2009	Amoxicillin, Clarithromycin	56	Cohort study	Cognitive function improvement	[89]
Howard et al., 2020	Minocycline	554	Randomized, double-blind, controlled trial	No effect on cognitive function	[94]
Dominy et al., 2019	COR388	573	Randomized, double-blind, controlled trial	NA	[95]

*NA* not available at this moment

### Treatment with antiviral agents

Two recent population studies conducted in Taiwan showed that antiviral treatment could help prevent dementia in patients with viral infections. The first showed that only 5.8% of HSV-1 and HSV-2 infected patients treated with anti-herpetic medications developed dementia over a 10-year follow-up period compared to 28.3% of untreated HSV-infected patients. Treatment of these HSV-1 and HSV-2 infected patients with the antiviral agent acyclovir, famciclovir, ganciclovir, valacyclovir and valganciclovir, either individually or in combination, reduced the risk of developing dementia [77]. The second study showed that treatment with antiviral agents reduced the risk of developing dementia by 45% in patients infected with herpes zoster compared to that for untreated infected patients [78]. Another interesting case is that of two siblings with chromosomally-integrated HHV-6A who suffered from cognitive difficulties. Several repeated courses of treatment with valganciclovir led to a near-complete clinical resolution in both patients [79]. The most generally promising drug for the treatment of herpetic viral infections appears to be valacyclovir, a prodrug of acyclovir. Valacyclovir was one of the first antivirals to enter into clinical trials against AD because of its high selectivity towards infected cells, favourable safety profile, and ability to enter the CNS. Its most obvious disadvantage is its narrow anti-herpetic effectivity; it is most potent against HSV-1 and HSV-2 [80, 81].

### Treatment with antibacterial agents

Antibiotics are very important drugs used to treat bacterial and fungal infections. The antibacterial agents most commonly investigated in the context of AD are doxycycline and rifampicin (rifampin). Twenty-eight years ago, Namba et al. reported an absence of senile plaques in leprosy patients who had undergone long-term treatment with rifampicin [82]. Twelve years later, Loeb et al. performed a controlled trial with 101 patients diagnosed with mild to moderate AD, who were randomly split into two groups. Over 3 months, one group received combined therapy with rifampin (300 mg) and doxycycline (200 mg), while the second group received a placebo [83]. Cognitive function evaluations revealed that the antibiotic-treated group exhibited significantly lower levels of cognitive decline after six months. Interestingly, both of these antibiotics also exhibit anti-amyloidogenic activity [84–87]. Balducci and Forloni also showed that doxycycline could abolish amyloid-β oligomer-mediated memory impairment and reduce neuroinflammation in mouse models of AD [88]. Kountouras et al. found that AD patients who received a successful triple eradication therapy with omeprazole, clarithromycin, and amoxicillin had better cognitive and functional results at a 2-year check-up than patients who did not receive such treatment [89]. Another antibiotic with promising anti-neuroinflammatory and the neuroprotective effect is minocycline [90–92]. In a mouse model of AD, minocycline reversed memory impairment caused by the administration of amyloid-β oligomers and reduced levels of the inflammatory cytokines L-1β, TNF-α, IL-4 and IL-10 in the brain and serum [93]. On the other hand, Howard et al. reported that minocycline did not delay the progress of cognitive or functional impairment in patients with mild AD over 2 years [94]. In addition to antibiotics, small-molecule inhibitors targeting gingipains, toxic proteases from *P. gingivalis*, have been developed [95]. One such compound, COR388, is currently being tested against AD in a Phase 2/3 clinical trial. In a recent study, aged dogs with oral infections of *P. gulae* and periodontal disease were treated with COR388 by oral administration. COR388 inhibited the lysine-gingipain target and reduced the *P. gulae* load in the saliva, buccal cells, and gingival crevicular fluid [96].

### Treatment with antifungal agents

Clinical trials with antifungal compounds were proposed by Alonso et al. [48]. Voriconazole, fluconazole, flucytosine and amphotericin B deoxycholate are antifungals with good CNS permeability that may be suitable for this purpose. In some cases, it may be beneficial to combine such treatments with neurosurgery, as noted in a recent review by Goralska et al. [97]. Combined therapies should also be considered for AD patients exhibiting signs of a multifungal infectious burden [51].

### Treatment with antiparasitic agents

Antiparasitic treatments targeting *Toxoplasma gondii* rely on two types of drugs, namely inhibitors of dihydrofolate reductase and dihydropteroate synthetase [98]. The first choice agent for treating neurotoxocariasis is likely to be albendazole, which exhibits good blood–brain permeability [99]. Because achieving efficient uptake of such drugs into tissues (particularly the brain) is very challenging, considerable efforts have been made to develop alternative derivatives, formulations, or delivery vehicles. Polyethylene glycol-conjugated and chitosan- or liposome-encapsulated compounds resulting from these efforts have demonstrated significant efficiency gains [100]. Albendazole combined with praziquantel is also an effective treatment for neurocysticercosis [101].

### Treatment with anti-biofilm agents

An important aspect of AD’s infection hypothesis is that some microorganisms can evade immune responses by various mechanisms, particularly by forming biofilms. Biofilms were first described by Costerton et al*.*, who observed clustering of bacteria in a polysaccharide matrix [102]. These structures are organized systems that protect microorganisms against stressful conditions and are formed by both bacteria and fungi [103]. Interestingly, viruses have also been shown to form biofilm-like assemblies [104]. Additionally, biofilms can be polymicrobial, allowing multiple microbe species to co-exist in one community [105]. For example, Mazaheritehrani et al. showed that *Candida* biofilms also shield HSV-1 viruses, which remain infective and releasable under this protection [106]. A subsequent study showed that this shelter protects HSV-1 against physical and chemical treatments, including laser and aciclovir or foscarnet therapy [107]. Coexistence of bacteria and fungi has also been reported [108]. In the context of AD pathology, some researchers have suggested that amyloid senile plaques in CNS tissues are biofilms [109, 110]. If so, biofilms are important therapeutic targets. This may also be true for *Toxoplasma gondii* because current treatments are effective against the active (tachyzoites) stage but ineffective against the latent cystic stage (bradyzoites) [98].

There are ongoing efforts to develop treatments targeting fungal and bacterial biofilms [111, 112] and *Toxoplasma* tissue cysts [113]. In addition to the compounds mentioned above, there is considerable interest in the opportunities offered by *N*-acetylcysteine, which was repeatedly found to have beneficial effects in the treatment of neurodegenerative diseases including AD [114]. Importantly, this compound exhibits strong activity against biofilms of both bacteria and *Candida* [115, 116]. Supportive treatments based on essential oils have also shown promise. For example, experimental studies performed by Feng et al. revealed that certain essential oils are highly effective against the stationary phase of *Borrelia burgdorferi* [117, 118] and various fungi [119].

## Conclusions

A growing number of research projects are probing the roles of pathogens in the development of AD. In the past, studies of this type focused mainly on individual pathogens [120, 121]. However, a growing body of evidence suggests that the aetiology of AD is driven at least in part by the coexistence of multiple pathogens. This insight may open up new ways of understanding, studying, and treating this disease, or even of preventing its onset altogether.

**Fig. 3:**
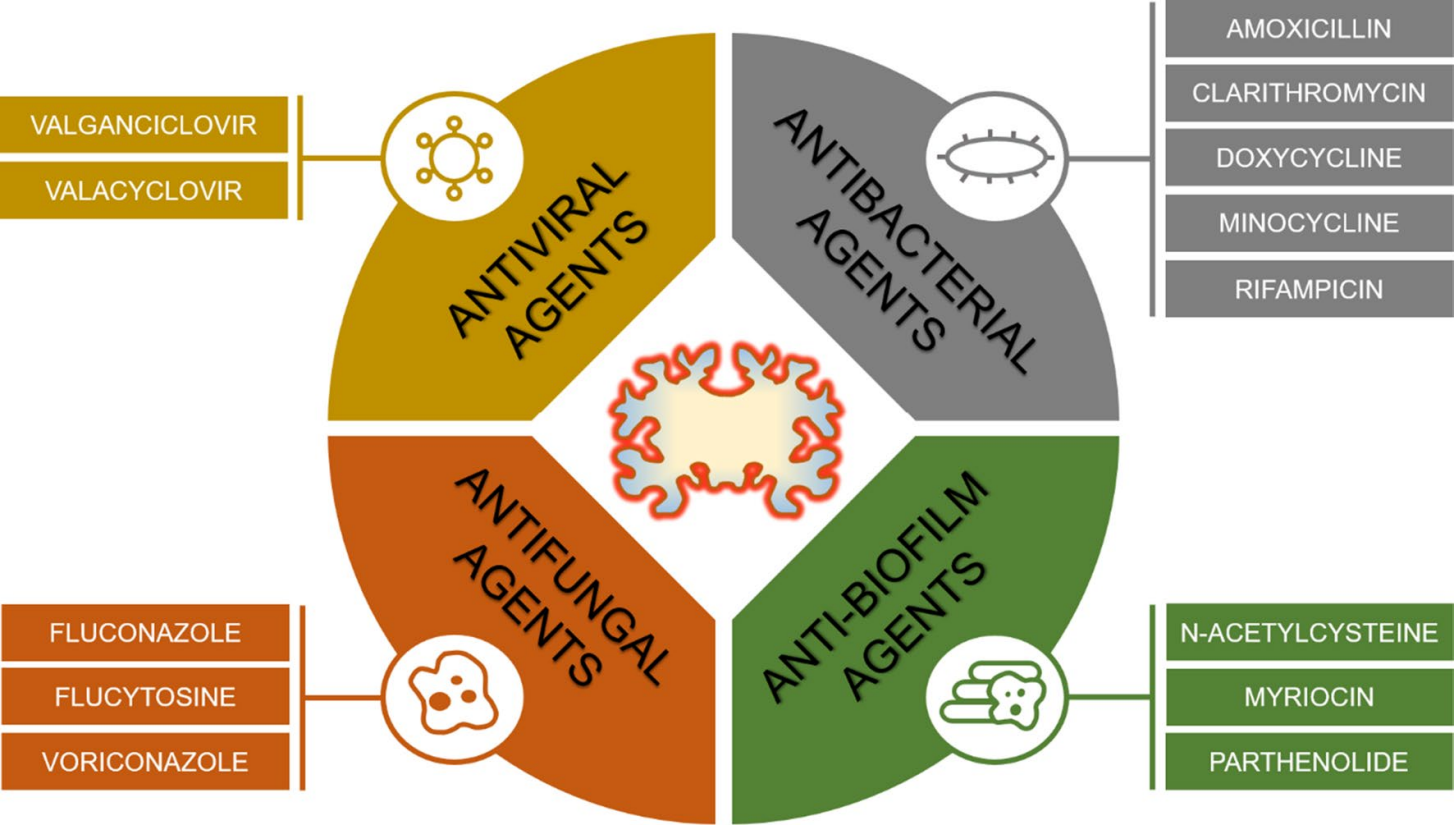
Potential antimicrobial treatment of patients with AD. The proposed therapeutic strategy consists of a combination of antiviral, antibacterial, antifungal, and anti-biofilm agents. Selected antimicrobial agents represent examples of potential therapeutics for the treatment of patients with AD. Degenerated brain tissue is represented by a yellow cartoon

From the standpoint of prevention, it is noteworthy that changes in brain functionality appear long before the onset of AD-induced cognitive dysfunction [122]. Moreover, various fungi and bacteria have been detected in disease-free control subjects [14, 51], and several studies have demonstrated connections between infectious burden and reduced cognitive function in adults [25, 66, 69]. This suggests a need for further research on screening for various pathogens in multiple matrices using a battery of diagnostic methods. The detection of specific pathogens or pathogen classes in middle-aged adults showing early signs of reduced cognitive function could then be followed by personalized preventative anti-microbial treatment (Fig. 3). Similar procedures could also be applied to patients already suffering from AD. Additionally, pathogens’ natural tendency to evade the immune system should be taken into account during diagnosis and when choosing treatments.

It is well established that the microbiomes of our bodies host vast microbial communities. These microbial communities communicate with each other internally, but they also communicate externally with the human host, affecting many metabolic processes [123]. They influence the immune system but also modulate the development of neural tissues in conjunction with neuromodulators and neurotransmitters. As a result, they can profoundly influence health [124]. The influence of changes in the gut microbiome on AD has been investigated [125, 126]. Several environmental factors, including antibiotic and antifungal treatments, can cause the development of a dysbiotic state within these communities [127, 128]. Mounting evidence indicates that gut dysbiosis may promote Aβ aggregation and neuroinflammation in AD development [129]. Broad-spectrum antimicrobials can be thus “two-edged swords”. Therefore, additional measures to optimize the gut microbiota composition, including probiotics, specific foods, and dietary patterns, should be taken into account when considering potential antimicrobial AD treatments.

Another recent discovery that could play an incredibly important role in diagnosing and treating AD, particularly when considering treatments targeting polymicrobial infections, is that the brain might have its unique microbiome [130]. This theory is supported by the results of Alonso et al., who demonstrated the presence of various bacterial and fungal species in both AD patients and healthy controls [14]. Further research on AD from the poly-microbial-inflammatory-microbiome point of view is therefore needed. The results of such studies may reveal a need for more personalized and complex ways of both diagnosing and treating the disease.

## Supplementary Information

**Additional file 1** Methodology of the literature search.

## Data Availability

Not applicable.
