# Supplementary material for: Multi-pathogen infections and Alzheimer’s disease
Source: Microb Cell Fact. 2021 Jan 28;20:25. doi: 10.1186/s12934-021-01520-7 (PMC7844946; doi:10.1186/s12934-021-01520-7)
Supplement: Supplementary file 1 — Additional file 1 Methodology of the literature search. [file 12934_2021_1520_MOESM1_ESM.docx]

**Additional file 1**

**Multi-pathogen Infections and Alzheimer’s Disease**

Dana Vigasova^1,2,#^, Michal Nemergut^2,#^, Barbora Liskova^2^, Jiri Damborsky^1,2*^

^1^ International Clinical Research Center, St. Anne’s University Hospital Brno, Pekarska 53, 656 91 Brno, Czech Republic; ^2^ Loschmidt Laboratories, Department of Experimental Biology and RECETOX, Faculty of Science, Masaryk University, Kamenice 5, 625 00 Brno, Czech Republic

# Shared first authors: Dana Vigasova – dana.vigasova09@gmail.com, Michal Nemergut – michal.nemergut@gmail.com; *Author for correspondence: Jiri Damborsky – jiri@chemi.muni.cz

**Methodology of the literature search**

A PubMed search was conducted using specific keywords to identify articles that describe Alzheimer`s disease (AD) and cognitive decline/impairment in the context of infectious burden. The included articles were required to contain at least two different pathogens in their study. The emphasis was mainly on experimental studies that included patients or samples from patients with AD.

Keywords used for literature search:

- Herpes simplex virus and Alzheimer`s disease/ Human herpes virus and Alzheimer`s disease
- HHV-2 and Alzheimer`s disease/HSV-2 and Alzheimer`s disease
- VZV and Alzheimer`s disease/ Varicella zoster virus and Alzheimer`s disease/ HHV-3 and Alzheimer`s disease
- HHV-4 and Alzheimer`s disease/ EBV and Alzheimer`s disease/ Epstein-Barr virus and Alzheimer`s disease
- CMV and Alzheimer`s disease/ Cytomegalovirus and Alzheimer`s disease/ HHV-5 and Alzheimer`s disease
- HHV-6 and Alzheimer`s disease
- HHV-7 and Alzheimer`s disease
- Periodontal bacteria and Alzheimer`s disease
- Spirochetes and Alzheimer`s disease
- *Porphyromonas gingivalis* and Alzheimer`s disease
- *Treponema* and Alzheimer`s disease (neurosyphilis excluded)
- *Borrelia burgdorferii* and Alzheimer`s disease
- *Chlamydia pneumoniae* and Alzheimer`s disease
- *Helicobacter pylori* and Alzheimer`s disease
- Fungal infections and Alzheimer`s disease
- *Toxoplasma gondii* and Alzheimer`s disease
- Parasites and Alzheimer`s disease
- Cysticercosis and Alzheimer`s disease
- Toxocarosis and Alzheimer`s disease
- Cysticercosis and Dementia
- Toxocarosis and Dementia
- Neurotoxocariasis
- Infectious burden and Alzheimer`s disease/ Infectious burden and cognitive decline/ Infectious burden and cognitive impairment
- Antibiotics therapy and Alzheimer`s disease
- Antiviral therapy and Alzheimer`s disease
- Antifungal therapy and Alzheimer`s disease
- N-Acetylcysteine and Alzheimer`s disease
